# Attenuation of antigen-induced airway hyperresponsiveness and inflammation in CXCR3 knockout mice

**DOI:** 10.1186/1465-9921-12-123

**Published:** 2011-09-22

**Authors:** Yi Lin, Haibo Yan, Yu Xiao, Hongmei Piao, Ruolan Xiang, Lei Jiang, Huaxia Chen, Kewu Huang, Zijian Guo, Wexun Zhou, Bao Lu, Jinming Gao

**Affiliations:** 1 Department of Respiratory Diseases, Peking Union Medical College Hospital, Chinese Academy of Medical Sciences & Peking Union Medical College, Beijing 100730, China; 2 Department of Respiratory Diseases, Yanbian University Affiliated Hospital, Yanbian, Jilin 133000, China; 3 Department of Pathology, Peking Union Medical College Hospital, Chinese Academy of Medical Sciences & Peking Union Medical College, Beijing 100730, China; 4 Department of Physiology and Pathophysiology, Peking University Health Sciences Center, Beijing 100088, China; 5 Department of Respiratory Medicine, Chaoyang Hospital, Capital University of Medical Sciences, Beijing 100023, China; 6 Ina Sue Perlmutter Laboratory, Children's Hospital, Harvard Medical School, Boston, MA 02115, USA

**Keywords:** chemokine receptor, CXCR3, CD8+ T lymphocyte, airway inflammation, airway hyperresponsiveness

## Abstract

**Background:**

CD8+ T cells participate in airway hyperresponsiveness (AHR) and allergic pulmonary inflammation that are characteristics of asthma. CXCL10 by binding to CXCR3 expressed preferentially on activated CD8+ T cells, attracts T cells homing to the lung. We studied the contribution and limitation of CXCR3 to AHR and airway inflammation induced by ovalbumin (OVA) using CXCR3 knockout (KO) mice.

**Methods:**

Mice were sensitized and challenged with OVA. Lung histopathological changes, AHR, cellular composition and levels of inflammatory mediators in bronchoalveolar lavage (BAL) fluid, and lungs at mRNA and protein levels, were compared between CXCR3 KO mice and wild type (WT) mice.

**Results:**

Compared with the WT controls, CXCR3 KO mice showed less OVA-induced infiltration of inflammatory cells around airways and vessels, and less mucus production. CXCR3 KO mice failed to develop significant AHR. They also demonstrated significantly fewer CD8+ T and CD4+ T cells in BAL fluid, lower levels of TNFα and IL-4 in lung tissue measured by real-time RT-PCR and in BAL fluid by ELISA, with significant elevation of IFNγ mRNA and protein expression levels.

**Conclusions:**

We conclude that CXCR3 is crucial for AHR and airway inflammation by promoting recruitment of more CD8+ T cells, as well as CD4+ T cells, and initiating release of proinflammatory mediators following OVA sensitization and challenge. CXCR3 may represent a novel therapeutic target for asthma.

## Introduction

Asthma is characterized by the persistence of chronic airway inflammation, which further leads to airway hyperresponsiveness (AHR), and mucus hypersecretion. Therefore, asthma treatment with inhaled corticosteroids (ICS) has been directed towards preventing and suppressing inflammation. Asthma control defined by international guidelines can be achieved and maintained by ICS alone or in combination with long-acting β_2 _agonist in the majority of asthma patients [1]. However, it is estimated that 5-10% of patients with difficult-to-treat asthma are refractory to the current therapies, and long-term use of ICS has been associated with side effects [2,3]. Therefore, searching for new pharmacological agents to meet these unmet clinical needs remains a priority objective [4].

A key step in the initiation and progression of asthma is the persistent recruitment of inflammatory cells into the airways of asthma patients in response to allergen, a process closely regulated by a variety of chemokines [5]. The expression of distinct chemokine receptors on infiltrating cell populations, especially on lymphocytes and eosinophils which are highly implicated in the pathogenesis of asthma, may represent a novel target for attenuating the influx of these inflammatory cells into the airways during the asthmatic process [6,7]. Because of the complexity of the promiscuous chemokine system [7], it has been difficult to identify the specific role of a single chemokine receptor in the asthmatic process.

Interferon-γ inducible CXCL10, one of CXCR3 ligands, is abundantly expressed in bronchiolar epithelial cells and airway smooth muscle cells of patients with asthma. Upon binding to its specific CXCR3 ligand preferentially expressed on activated CD8+ T cells and eosinophils [8,9], CXCL10 is a chemoattractant for activated T-cells and eosinophils into the inflamed sites [7,9,10]. CXCL10 transgenic mice exhibited airway hyperresponsiveness in an OVA-sensitized model [11]. An interaction of CXCL10/CXCR3 has been reported to contribute to the migration of mast cells into airway smooth muscle in asthma [3]. Increased numbers of CXCR3+ T cells in blood have been reported to be associated with asthma severity [12]. Furthermore, a two-week course of oral prednisolone did not change the number of peripheral blood CXCR3+ T cells in asthma patients [13]. Recently, a small-molecule antagonist for both CXCR3 and CCR5 has been reported to alleviate some asthmatic responses after antigen exposure, such as AHR and lung inflammation [14]. Taken together, these findings indicate that CXCR3/CXCL10 axis may play a pivotal role in the pathogenesis of asthma through recruitment of T cells, as well as other inflammatory cells, into airways and lung parenchyma.

Elucidation of the precise role of CXCR3 in asthma has been facilitated by the generation of CXCR3 knockout (KO) mice. In this study, we investigated the specific contribution of CXCR3 in a model of ovalbumin (OVA)-induced asthma using CXCR3 KO mice and WT mice as control.

## Materials and methods

### Mouse model of OVA-induced airway inflammation

Mice line depleted of CXCR3 gene has been established by gene targeting as described elsewhere [15]. CXCR3 KO mice (kindly gifted by Dr. Gerard, Harvard University) and WT mice (Experimental Animal Research Center, Beijing, China) with C57BL/6 background (backcrossed for more than 14 generations), were maintained in a pathogen-free mouse facility at Peking Union Medical College Animal Care Center. Clean food and water were supplied with free access. Gender-matched mice aged 10-12 weeks (~20-22 grams of weight) were used in the experiments.

Mice were given intraperitoneal injection on days 0 and 14 with 50 μg of OVA (Grade V, Sigma, MO) absorbed to 2.25 mg Alum (Pierce) in 200 μl of sterile saline. Ten days after the last sensitization, mice were challenged with 1% aerosolized OVA for 20 minutes on six consecutive days in a chamber using a PARI nebulizer. Sham mice received aluminum hydroxide and were exposed to 0.9% NaCl solution alone using the same protocol. Mice were sacrificed 24 hours after the last aerosol challenge

All experiments were performed according to international and institutional guidelines for animal care, and approved by Peking Union Medical College Hospital Ethics Committee for animal experimentation.

### Histological analysis of lung tissue

The mice were sacrificed and the lungs were removed, inflated to 25 cmH_2_O with 10% formalin and fixed overnight, then embedded in paraffin, and sectioned at 5 μm as described previously [16-18]. Lung sections were stained with hematoxylin & eosin reagent. An index of histopathological change was evaluated by scoring the severity and extent of the infiltration of inflammatory cells around airways and vessels, and epithelial thickening according to previously published methods [14,19,20]. Periodic acid-Schiff reagent was used to stain the mucus-staining cells. The pathological analysis was independently performed in each mouse by two pathologists blinded to the genotype.

### Bronchoalveolar lavage (BAL)

24 hours after the final aerosol challenge, mice were killed and the trachea was cannulated by using 20-gauge catheter. BAL was performed three times with 0.8 mL of ice-cold PBS (pH 7.4) each. The BAL fluid was spun at 1500 rpm for 5 min at 4°C, and supernatant was collected and stored at -70°C until analyzed.

### Labeling cells from BAL fluid

50 uL of 2 × 10^7^/ml of cells recovered from BAL fluid was used. 10 μL of blocking buffer was added to the cells for 15 min on ice. After washing, cells were then incubated with 50 μL of FITC-conjugated anti-CD4 Ab and PE-conjugated anti-CD8 Ab or control mouse IgG2b (BD PharMingen, San Diego, CA) for 1 hr on ice. Cells were washed by PBS and fixed in PBS containing 2% formalin. Cells were subjected to flow cytometer using a FACScan (Beckman Coulter, Germany) [16].

### Determination of protein content in BAL Fluid

Total protein content in BAL fluid was assayed using the BCA Protein Assay Kit (Thermo Fisher Scientific, China) according to manufacturer's instructions.

### ELISA analysis of IL-4, IFNγ, and CXCL10 in BAL fluid

The concentrations of IL-4, IFNγ, and CXCL10 in BAL fluid were determined by ELISA kits (R&D systems) according to manufacturer's recommendations.

### Extraction of total RNA and quantitative real-time PCR and analysis

Total RNA was extracted from whole lung using guanidine isothiocyanate methods and reverse-transcribed to cDNA using Omniscript Reverse Transcriptase (QIAGEN, Hilden, Germany). Quantitative real-time RT-PCR amplification and analysis were carried out by using ABI Prism 7700 sequence detector system (Perkin Elmer, Germany). PCR was carried out with the TaqMan Universal PCR Master Mix (PE Applied Biosystems) using 1 μL of cDNA in a 20 μL final reaction volume.

### Airway responsiveness

Airway responsiveness to inhaled methacholine (Mch) was determined in mice 24 hours after the final aerosol challenge. Airway resistance (RL) was

assessed as previously described for invasive analysis of lung mechanics using a computer-controlled small animal ventilator, Flexivent system (Scireq, Montreal, PQ, Canada) [16,17]. Changes in tracheal pressure were measured in response to challenge with saline, followed by increasing concentrations of methacholine (3.125, 6.25, 12.5, and 25 mg/ml).

### Statistics

Data are expressed as means ± SEM. Comparisons were carried out using one-way ANOVA followed by unpaired Student's *t *test (Graph Pad Software Inc., San Diego, CA). A value of *P *less than 0.05 was considered significant.

## Results

### Airway inflammation in OVA-sensitized and -exposed mice

**Figure 1:**
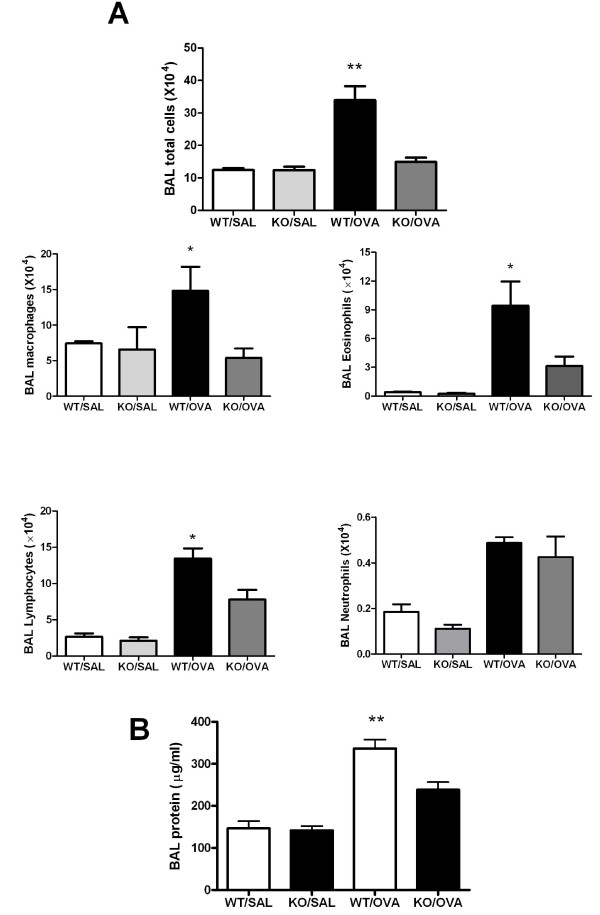
**Difference of airway inflammation between CXCR3-deficient mice and the WT controls**. Mice were sensitized and challenged with OVA as described in Material and Methods. A, Total inflammatory cells and differential subpopulations in BAL fluid, *n *= 6-8 animals per group, *, *p *< 0.05 vs other groups, **, *p *< 0.01 vs other groups. B, Protein concentrations in BAL fluid, *n *= 4-6 animals per group, *, *p *< 0.05 vs other groups.

To determine whether CXCR3 depletion affects the antigen-induced infiltration of inflammatory cells into airways, we estimated the cell subpopulations in BAL fluid following antigen sensitization and challenge. There was significantly less infiltration of total inflammatory cells, eosinophils, lymphocytes, and macrophages into airways in OVA-sensitized and -challenged CXCR3 KO than in similarly treated-WT mice (Figure 1A). The total protein content in BAL fluid, an index of permeability of the endothelial-capillary barrier, was significantly higher in OVA-sensitized and challenged WT mice than in CXCR3 KO mice (Figure 1B).

### Semiqualitative analysis of inflammation in the lung by histopathology

The histopathology of lungs from CXCR3 KO and WT mice after with or without OVA induction was reviewed by a pathologist blinded to the origin of the tissue and genotypes. We assessed the tissue for inflammation around bronchus and vessel areas, epithelial thickening, and mucous hypersecretion. There were no inflammatory response around bronchial and vascular spaces, and no mucus hypersecretion in sham mice (data not shown).

**Figure 2:**
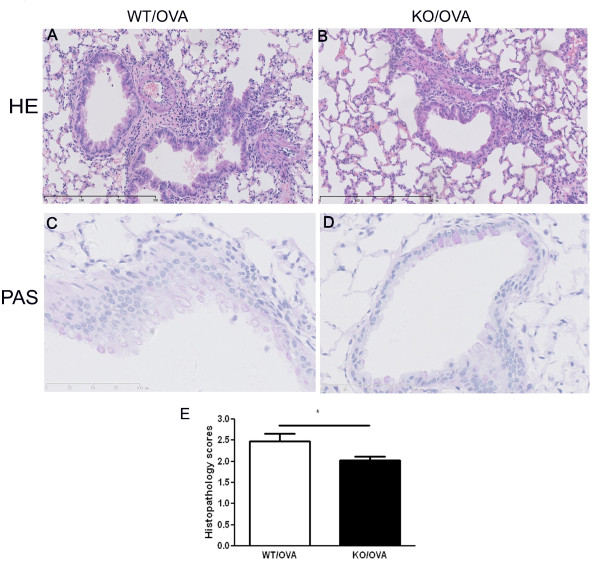
**Effect of CXCR3 on airway inflammation and mucous production**. Mice were immunized and challenged with OVA. Lungs were analyzed by H&E staining, scored for histopathological changes in lung inflammation and thickened airway epithelium, and by periodic acid-Schiff-staining for mucous production as described in *Materials and Methods*. A-D, Representative photomicrographs of hematoxylin- & eosin and periodic acid-Schiff-stained lung tissues. E, Semiquantative analysis of histopathologic changes using a scoring method as described in Materials and Methods, *n *= 5 animals per group, *, *p *< 0.05.

Compared with similarly-treated CXCR3 KO mice, OVA-sensitized and challenged WT mice showed the typical pathological characteristics of allergic pulmonary inflammation evidenced by thickened airway epithelium and more inflammatory cells in the peribronchial area and around vessles, in which the predominant cell types were macrophages, lymphocytes, and eosinophils (Figure 2A and 2B). Consistent with lack of significant inflammation in the airways, CXCR3 KO mice did not produce obvious mucus secretion in the larger airways, whereas WT mice had mucus hypersecretion in their lungs (Figure 2C and 2D).

We semi-quantitatively scored the histopathological findings. There was a significant increase in inflammation scores in WT mice compared with CXCR3 KO mice (2.48 ± 0.17 vs 2.02 ± 0.09, P = 0.045) (Figure 2E).

Although immunization and aerosol challenge with OVA induced the elevation of total IgE and OVA-specific-IgE in serum from both WT and CXCR3 KO mice compared with the sham mice, there was no significant difference in total IgE and OVA-specific IgE between WT mice and CXCR3 KO mice (data not shown).

### OVA-induced AHR

**Figure 3:**
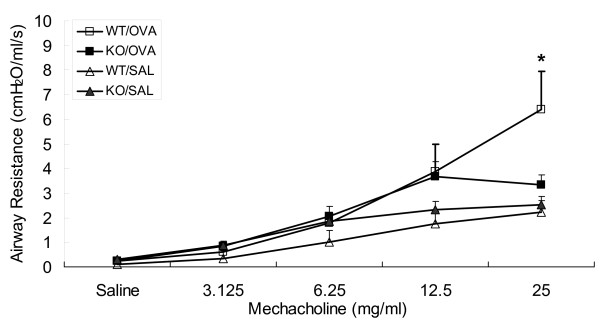
**Effect of CXCR3 deficiency on airway responsiveness to inhaled methacholine**. Immunized and challenged WT mice showed significantly higher airway responsiveness at 25 mg/ml mechacholine than all other groups as determined by one-way ANOVA, *n *= 8-12 animals per group. Note that airway responsiveness was significantly higher in immunized and challenged WT mice compared with the similarly-treated CXCR3 KO mice as determined by unpaired t-test (p < 0.05).

AHR is an endpoint of airway inflammation, and one of key characteristics of asthma. Previous data has shown that blockade of CXCR3 and CCR5 using a synthetic small-molecule compound can significantly attenuate antigen-induced AHR, as well as allergic pulmonary inflammation [14]. We further addressed this question by using CXCR3 KO mice. As shown in Figure 3, one-way ANOVA demonstrated that sensitized and challenged WT mice developed significant increases in lung resistance in response to increasing doses of inhaled methacholine. However, sensitized and challenged CXCR3 KO mice did not develop significant increases in lung resistance in response to methacholine compared with challenged but not sensitized control mice. Particularly, airway responsiveness was significantly higher in immunized and challenged WT mice compared with the similarly-treated CXCR3 KO mice as determined by unpaired t-test (p < 0.05).

### OVA-induced infiltration of CD8+T cells in airways

**Figure 4:**
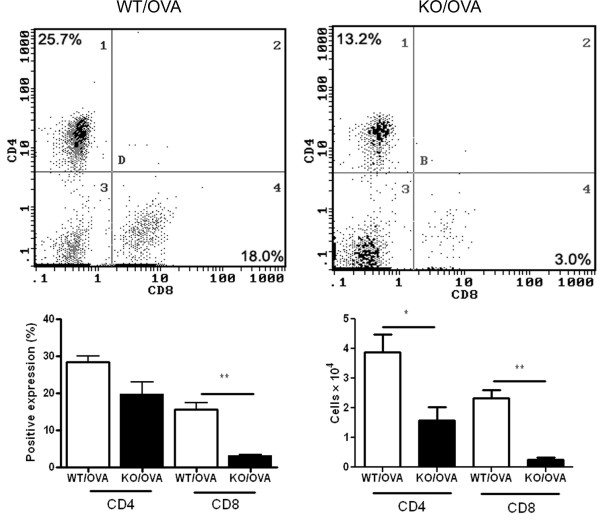
**Effect of OVA sensitization and exposure on CD8+ and CD4+ T cell infiltration into airways in CXCR3 KO and WT mice**. Top panel, representative histogram showing expression of CD4+ T cells and CD8+ T cells in BAL fluid. The data presented are from one representative of four independent experiments. Bottom panel, pooled data showing the percentage and aboslute number of CD4+ T cells and CD8+ T cells in BAL fluid, *n *= 4 separate experiments, *, *p < 0.05*, **, *p *< 0.01.

The percentage and absolute numbers of CD8+ T cells in BAL fluid from CXCR3 KO mice were significantly decreased compared to that from WT mice after antigen sensitization and exposure (3.3 ± 0.3% vs 15.6 ± 1.9%, p = 0.003; 0.3 ± 0.1 × 10^4 ^vs 2.3 ± 0.3 × 10^4^, p = 0.002) (Figure 4). The percentage of CD4+ T cells was not statistically higher in BAL fluid recovered from WT mice than from CXCR3 KO mice (28.5 ± 1.5% vs 19.8 ± 1.3%, p = 0.07), however, the absolute number of CD4+ T cells was significantly decreased in CXCR3 KO mice (3.9 ± 0.6 × 10^4 ^vs 1.6 ± 0.5 × 10^4^, p = 0.037) (Figure 4). These data demonstrate that trafficking of CD8+ T cells, as well as CD4+ T cells, to the airways induced by OVA was impaired by the absence of CXCR3.

### mRNA expression of cytokines

**Figure 5:**
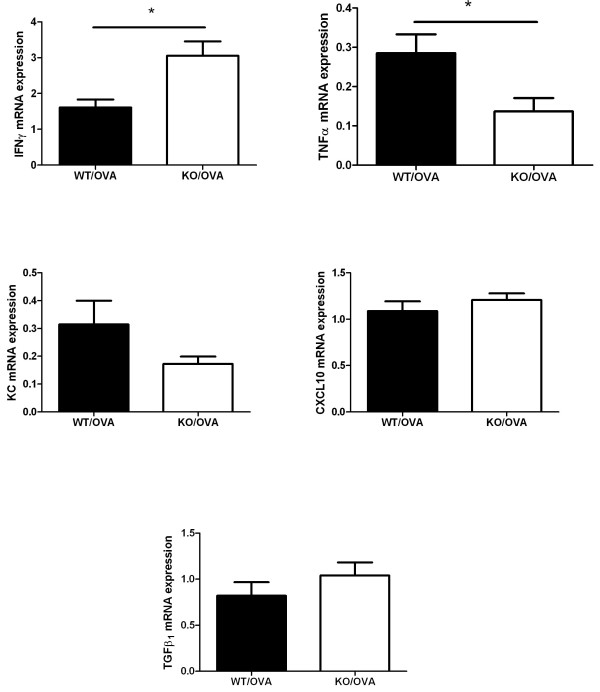
**Effect of CXCR3 deficiency on mRNA expression of cytokines in lung measured by RT-PCR**. Lungs were collected at 24 hours after last aerosol challenge from similarly treated-CXCR3 KO mice and WT mice, *n *= 4-5 mice per group, *, *p *< 0.05.

The expression of IFNγ mRNA in lungs by quantitative real-time PCR was significantly inhibited in response to OVA immunization and challenge in WT mice, but not in CXCR3 KO mice. By contrast, mRNA expression of TNFα in lung was significantly reduced in CXCR3 KO mice (Figure 5). We did not find any difference in mRNA expression of the other cytokines, including CXCL10, KC, and TGFβ1 (Figure 5). The mRNA expression of these cytokines was significantly lower in sham mice in comparison with OVA-immunized and challenged mice of both mouse genotypes (data not shown).

### Cytokine concentrations in BAL fluid

**Figure 6:**
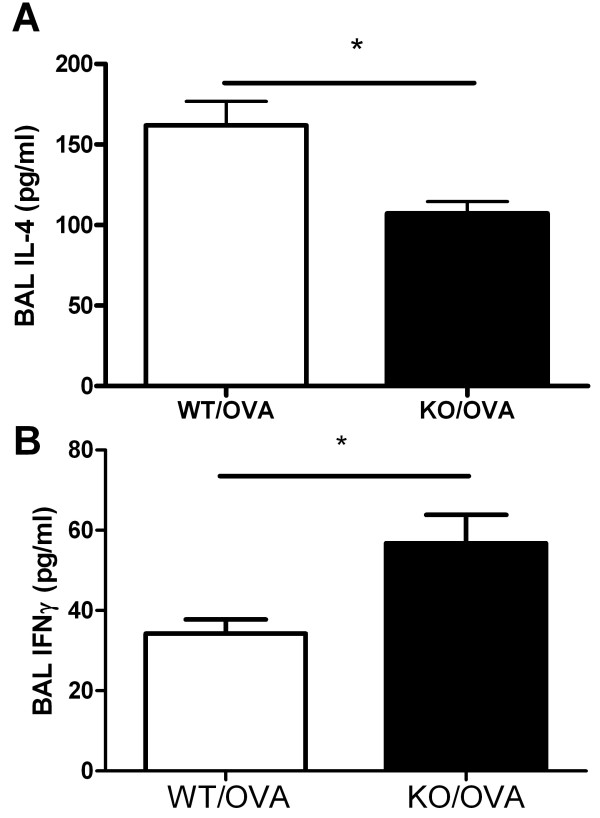
**Effect of CXCR3 deficiency on production of cytokines in BAL fluid assessed by ELISA**. BAL fluids were recovered from similarly treated-CXCR3 KO and WT mice, *n *= 4-6 mice per group, *, *p *< 0.05.

IL-4 concentration in BAL fluid was significantly higher in OVA-immunized and challenged WT mice than that in similarly treated-CXCR3 KO mice (Figure 6A), whereas the level of IFNγ in BAL fluid was significantly higher in CXCR3 KO mice than in WT mice (Figure 6B). CXCL10 concentration in BAL fluid was similarly elevated between CXCR3 KO mice and WT mice after induction of OVA (data not shown). The concentrations of these cytokines in BAL fluid by ELISA were undetectable.

## Discussion

To the best of our knowledge, this is the first report demonstrating an important role of CXCR3 in regulating airway responsiveness and allergic airway inflammation by using mice with targeted deletion of CXCR3 gene in animal model. In OVA-sensitized and exposed CXCR3 KO mice, we observed: [1] a significant reduction in the severity of allergic airway inflammation as evidenced by fewer inflammatory cells (particularly less CD8+ T cells, as well as CD4+ T cells) in the airways, significantly less protein leakage, and a reduction in mucus production and [2] significantly decreased AHR. Therefore, CXCR3 may have a direct inhibition of infiltration of inflammatory cells associated with the asthmatic response and furthermore, on the development of AHR. Our data are consistent with previous reports that also support the importance of CXCR3 in the initiation and progression of airway inflammation in asthma [12,21,22]. Thus, the increased numbers of CXCR3+ T cells in blood was reported to be associated with asthma severity [12]. Data from mouse models of asthma suggest that increases in recruitment of CXCR3+ T cells homing to the lung may increase the severity of asthmatic response [11]. Thus, blockade of CXCR3 may represent a novel target for asthma treatment.

AHR is a key component of the murine model of asthma. We showed that AHR was significantly abrogated in CXCR3 KO mice compared with the WT controls. Our data demonstrated significantly less CD8+ T cells, as well as CD4+ T cells, infiltrating airways of CXCR3 KO mice that were immunized and challenged with OVA. The explanation for the relative difference in infiltration of CD8+ T and CD4+ T cells into the airways between CXCR3 KO and WT mice in this model may partly be attributed to the downstream effect of CXCR3 activation. The association between CD8+ T cells and AHR has been reported previously [23,24]. Mice lacking CD8+ T cells failed to develop AHR and airway inflammation, suggesting a critical role for CD8+ T cells in the asthmatic responses [7,8]. The mechanism by which CD8+ T cells mediates AHR and allergic inflammation of airway may be due to accumulation of effector CD8+ T cells and CD4+ IL4+ T cells in the lung tissue [25,26]. Moreover, CD8+ T cells appear to be essential for the influx of eosinophils into the lung in respiratory virus infected mice [27]. Our data also showed less infiltration of CD4+ T cells into lungs of CXCR3 KO mice after OVA induction. Consistent with our results, the previous studies have demonstrated that CD4+ cells are required for eosinophilic lung inflammation in murine models of acute and chronic Th2-driven airway inflammation [28,29]

The allergic inflammation of airways induced by OVA is characterized by an increased number of Th2 cells, that secrete Th2-type cytokines. IL-4, one of key Th2-type cytokines, is highly relevant to the pathogenesis of asthma [26,30]. IL-4 has also been shown to be important for the functional activation of CD8+ T cells for the subsequent development of AHR and airway inflammation during the sensitization phase in a murine model [26]. Consistent with this study, we did find a significant elevation of IL-4 in the BAL fluid in OVA-sensitized- and challenged WT mice; however, such an elevation was substantially inhibited in similarly treated-CXCR3 KO mice. There is evidence supporting the presence of Th2-like CD8+ T cells that produce IL-4 and IL-5, not IFNγ [31]. Our data also demonstrated that more IL-4-producing CD4+ T cells were significantly infiltrating the airways of OVA-immunized and challenged WT mice than in similarly-treated CXCR3 KO mice. IL-4 is important in regulating IgE synthesis. However, there was no difference in total IgE and OVA-specific IgE in serum between both mouse genotypes. It is possible that other cytokines such as IL-13 are involved in the induction of IgE production in our model [32].

We also showed that induction of mRNA expression of pro-inflammatory cytokine TNFα in the lungs was significantly less in OVA-sensitized and challenged CXCR3 KO mice than that in OVA-sensitized and challenged WT mice. This might be due to the reduced accumulation of inflammatory cells in airways in CXCR3 KO mice, such as macrophages and CD4+ T cells, because there is evidence showing that monocytes and CD4+ T cells have the capability to produce TNFα [4].

There is evidence supporting an inhibitory effect of IFNγ on the full development of AHR [33-36]. In supporting these observations, we demonstrated that IFNγ at both mRNA and protein levels was significantly lower in OVA-sensitized and challenged WT mice than in similarly treated CXCR3 KO mice. IFNγ has been shown to inhibit the production of Th2-cytokines (IL-4, IL-5, and IL-13) from antigen-primed T-cells, partly by skewing toward Th1-type cells [33]. However, our data are somewhat inconsistent with the point that CXCL10-CXCR3 interaction has been known to promote Th1 other than Th2 inflammation. However, the allergen-induced asthmatic phenotype is not due to a single chemokine receptor, but other chemokine receptors, such as CCR5 and CCR6, expressed on inflammatory cells are also likely to be involved [21,37]. CCR5 preferentially expressed on Th1 cells has been shown to be upregulated upon OVA sensitization and exposure [14]. A small compound antagonizing both CCR5 and CXCR3 has been shown to decrease Th1-like airway inflammation in OVA-primed and exposed mice [14].

The observations presented in this study point to an important role for CXCR3 in a murine allergic model of asthma. However, it should be pointed out that CXCR3 KO mice showed only partial protection against OVA-induced AHR and airway inflammation. Further studies should be performed to determine how multiple chemokine receptors expressed on inflammatory cells and lung resident cells coordinately interact in a complex network to contribute to asthma pathogenesis. Because several chemokines share a single receptor, blockade of the chemokine receptor may represent a more effective way to inhibit the effect of multiple chemokines than blocking their production [5,38].

## Conclusion

In conclusion, our study shows that CXCR3 regulates OVA-induced allergic airway inflammation via recruitment of CD8+ T cells into the airways to trigger the release of proinflammatory cytokines including TNFα and IL-4 and inhibit the production of antiinflammatory mediators exemplified by IFNγ. Our findings suggest that designing an inhibitor specially targeting CXCR3 may be helpful for the treatment of asthma.

## Conflict of interest statement

None of the authors has a financial relationship with a commercial entity that has an interest in the subject of this manuscript.

## Authors' contributions

YL, HY and RX performed the whole experiment; YX carried out the pathological analysis, WZ facilitated the pathological analysis; HP, LJ, HC and ZG helped and did some experiments; KH performed the lung function assay; BL and JG designed and supervised the experiments, and drafted the manuscript. All authors have read and approve the final version of this manuscript.

## References

[B1] Bateman ED Hurd SS Barnes PJ Bousquet J Drazen JM FitzGerald M Gibson P Ohta K O'Byrne P Pedersen SE Global strategy for asthma management and prevention: Gina executive summary Eur Respir J 2008 31 1 143 178 10.1183/09031936.00138707 18166595

[B2] Holgate ST Polosa R The mechanisms, diagnosis, and management of severe asthma in adults Lancet 2006 368 9537 780 793 10.1016/S0140-6736(06)69288-X 16935689

[B3] Chanez P Wenzel SE Anderson GP Anto JM Bel EH Boulet LP Brightling CE Busse WW Castro M Dahlen B Severe asthma in adults: What are the important questions? J Allergy Clin Immunol 2007 119 6 1337 1348 10.1016/j.jaci.2006.11.702 17416409

[B4] Brightling C Berry M Amrani Y Targeting tnf-alpha: A novel therapeutic approach for asthma J Allergy Clin Immunol 2008 121 1 5 10 quiz 11-12 10.1016/j.jaci.2007.10.028 18036647 PMC3992375

[B5] Barnes PJ The cytokine network in asthma and chronic obstructive pulmonary disease J Clin Invest 2008 118 11 3546 3556 10.1172/JCI36130 18982161 PMC2575722

[B6] D'Ambrosio D Panina-Bordignon P Sinigaglia F Chemokine receptors in inflammation: An overview J Immunol Methods 2003 273 1-2 3 13 10.1016/S0022-1759(02)00414-3 12535793

[B7] D'Ambrosio D Mariani M Panina-Bordignon P Sinigaglia F Chemokines and their receptors guiding t lymphocyte recruitment in lung inflammation Am J Respir Crit Care Med 2001 164 7 1266 1275 11673221 10.1164/ajrccm.164.7.2103011

[B8] Loetscher M Gerber B Loetscher P Jones SA Piali L Clark-Lewis I Baggiolini M Moser B Chemokine receptor specific for ip10 and mig: Structure, function, and expression in activated t-lymphocytes J Exp Med 1996 184 3 963 969 10.1084/jem.184.3.963 9064356 PMC2192763

[B9] Jinquan T Jing C Jacobi HH Reimert CM Millner A Quan S Hansen JB Dissing S Malling HJ Skov PS Cxcr3 expression and activation of eosinophils: Role of ifn-gamma-inducible protein-10 and monokine induced by ifn-gamma J Immunol 2000 165 3 1548 1556 10903763 10.4049/jimmunol.165.3.1548

[B10] Liu LY Jarjour NN Busse WW Kelly EA Chemokine receptor expression on human eosinophils from peripheral blood and bronchoalveolar lavage fluid after segmental antigen challenge The Journal of allergy and clinical immunology 2003 112 3 556 562 10.1016/S0091-6749(03)01798-6 13679815

[B11] Medoff BD Sauty A Tager AM Maclean JA Smith RN Mathew A Dufour JH Luster AD Ifn-gamma-inducible protein 10 (cxcl10) contributes to airway hyperreactivity and airway inflammation in a mouse model of asthma J Immunol 2002 168 10 5278 5286 11994485 10.4049/jimmunol.168.10.5278

[B12] Kurashima K Fujimura M Myou S Ishiura Y Onai N Matsushima K Asthma severity is associated with an increase in both blood cxcr3+ and ccr4+ t cells Respirology (Carlton, Vic 2006 11 2 152 157 10.1111/j.1440-1843.2006.00822.x 16548899

[B13] Kurashima K Fujimura M Myou S Kasahara K Tachibana H Amemiya N Ishiura Y Onai N Matsushima K Nakao S Effects of oral steroids on blood cxcr3+ and ccr4+ t cells in patients with bronchial asthma Am J Respir Crit Care Med 2001 164 5 754 758 11549528 10.1164/ajrccm.164.5.2008132

[B14] Suzaki Y Hamada K Nomi T Ito T Sho M Kai Y Nakajima Y Kimura H A small-molecule compound targeting ccr5 and cxcr3 prevents airway hyperresponsiveness and inflammation Eur Respir J 2008 31 4 783 789 10.1183/09031936.00111507 18094012

[B15] Hancock WW Lu B Gao W Csizmadia V Faia K King JA Smiley ST Ling M Gerard NP Gerard C Requirement of the chemokine receptor cxcr3 for acute allograft rejection The Journal of experimental medicine 2000 192 10 1515 1520 10.1084/jem.192.10.1515 11085753 PMC2193193

[B16] Nie L Xiang R Zhou W Lu B Cheng D Gao J Attenuation of acute lung inflammation induced by cigarette smoke in cxcr3 knockout mice Respir Res 2008 9 82 10.1186/1465-9921-9-82 19087279 PMC2654035

[B17] Nie L Xiang RL Liu Y Zhou WX Jiang L Lu B Pang BS Cheng DY Gao JM Acute pulmonary inflammation is inhibited in cxcr3 knockout mice after short-term cigarette smoke exposure Acta pharmacologica Sinica 2008 29 12 1432 1439 10.1111/j.1745-7254.2008.00899.x 19026162

[B18] Nie L Liu ZJ Zhou WX Xiang RL Xiao Y Lu B Pang BS Gao JM Chemokine receptor cxcr3 is important for lung tissue damage and airway remodeling induced by short-term exposure to cigarette smoking in mice Acta Pharmacol Sin 31 4 436 442 10.1038/aps.2009.192 PMC4007663 20208554

[B19] Medoff BD Okamoto Y Leyton P Weng M Sandall BP Raher MJ Kihara S Bloch KD Libby P Luster AD Adiponectin deficiency increases allergic airway inflammation and pulmonary vascular remodeling Am J Respir Cell Mol Biol 2009 41 4 397 406 10.1165/rcmb.2008-0415OC 19168697 PMC2746986

[B20] Wittke A Weaver V Mahon BD August A Cantorna MT Vitamin d receptor-deficient mice fail to develop experimental allergic asthma J Immunol 2004 173 5 3432 3436 15322208 10.4049/jimmunol.173.5.3432

[B21] Thomas SY Banerji A Medoff BD Lilly CM Luster AD Multiple chemokine receptors, including ccr6 and cxcr3, regulate antigen-induced t cell homing to the human asthmatic airway J Immunol 2007 179 3 1901 1912 17641057 10.4049/jimmunol.179.3.1901

[B22] Campbell JD Gangur V Simons FE HayGlass KT Allergic humans are hyporesponsive to a cxcr3 ligand-mediated th1 immunity-promoting loop Faseb J 2004 18 2 329 331 14657006 10.1096/fj.02-0908fje

[B23] Betts RJ Kemeny DM Cd8+ t cells in asthma: Friend or foe? Pharmacol Ther 2009 121 2 123 131 10.1016/j.pharmthera.2008.09.001 18940198

[B24] Gelfand EW Dakhama A Cd8+ t lymphocytes and leukotriene b4: Novel interactions in the persistence and progression of asthma J Allergy Clin Immunol 2006 117 3 577 582 10.1016/j.jaci.2005.12.1340 16522456

[B25] Miyahara N Swanson BJ Takeda K Taube C Miyahara S Kodama T Dakhama A Ott VL Gelfand EW Effector cd8+ t cells mediate inflammation and airway hyper-responsiveness Nat Med 2004 10 8 865 869 10.1038/nm1081 15258576

[B26] Koya T Miyahara N Takeda K Matsubara S Matsuda H Swasey C Balhorn A Dakhama A Gelfand EW Cd8+ t cell-mediated airway hyperresponsiveness and inflammation is dependent on cd4+il-4+ t cells J Immunol 2007 179 5 2787 2796 17709492 10.4049/jimmunol.179.5.2787

[B27] Schwarze J Cieslewicz G Joetham A Ikemura T Hamelmann E Gelfand EW Cd8 t cells are essential in the development of respiratory syncytial virus-induced lung eosinophilia and airway hyperresponsiveness J Immunol 1999 162 7 4207 4211 10201948

[B28] Doherty TA Soroosh P Broide DH Croft M Cd4+ cells are required for chronic eosinophilic lung inflammation but not airway remodeling American journal of physiology 2009 296 2 L229 235 19060225 10.1152/ajplung.90543.2008 PMC2643996

[B29] Gavett SH Chen X Finkelman F Wills-Karp M Depletion of murine cd4+ t lymphocytes prevents antigen-induced airway hyperreactivity and pulmonary eosinophilia American journal of respiratory cell and molecular biology 1994 10 6 587 593 8003337 10.1165/ajrcmb.10.6.8003337

[B30] Schwarze J Cieslewicz G Joetham A Ikemura T Makela MJ Dakhama A Shultz LD Lamers MC Gelfand EW Critical roles for interleukin-4 and interleukin-5 during respiratory syncytial virus infection in the development of airway hyperresponsiveness after airway sensitization Am J Respir Crit Care Med 2000 162 2 Pt 1 380 386 10934057 10.1164/ajrccm.162.2.9903057

[B31] Cho SH Stanciu LA Holgate ST Johnston SL Increased interleukin-4, interleukin-5, and interferon-gamma in airway cd4+ and cd8+ t cells in atopic asthma Am J Respir Crit Care Med 2005 171 3 224 230 15502111 10.1164/rccm.200310-1416OC

[B32] Wills-Karp M Interleukin-13 in asthma pathogenesis Immunol Rev 2004 202 175 190 10.1111/j.0105-2896.2004.00215.x 15546393

[B33] Lack G Renz H Saloga J Bradley KL Loader J Leung DY Larsen G Gelfand EW Nebulized but not parenteral ifn-gamma decreases ige production and normalizes airways function in a murine model of allergen sensitization J Immunol 1994 152 5 2546 2554 8133062

[B34] Lack G Bradley KL Hamelmann E Renz H Loader J Leung DY Larsen G Gelfand EW Nebulized ifn-gamma inhibits the development of secondary allergic responses in mice J Immunol 1996 157 4 1432 1439 8759723

[B35] Koya T Takeda K Kodama T Miyahara N Matsubara S Balhorn A Joetham A Dakhama A Gelfand EW Rantes (ccl5) regulates airway responsiveness after repeated allergen challenge Am J Respir Cell Mol Biol 2006 35 2 147 154 10.1165/rcmb.2005-0394OC 16528011 PMC2643254

[B36] Dow SW Schwarze J Heath TD Potter TA Gelfand EW Systemic and local interferon gamma gene delivery to the lungs for treatment of allergen-induced airway hyperresponsiveness in mice Hum Gene Ther 1999 10 12 1905 1914 10.1089/10430349950017266 10466624

[B37] Mikhak Z Fukui M Farsidjani A Medoff BD Tager AM Luster AD Contribution of ccr4 and ccr8 to antigen-specific t(h)2 cell trafficking in allergic pulmonary inflammation J Allergy Clin Immunol 2009 123 1 67 73 e63 10.1016/j.jaci.2008.09.049 19062085 PMC2782398

[B38] Barnes PJ New therapies for asthma: Is there any progress? Trends Pharmacol Sci 31 7 335 343 10.1016/j.tips.2010.04.009 20554041

